# Effects of tailored neck-shoulder pain treatment based on a decision model guided by clinical assessments and standardized functional tests. A study protocol of a randomized controlled trial

**DOI:** 10.1186/1471-2474-13-75

**Published:** 2012-05-20

**Authors:** Martin Björklund, Mats Djupsjöbacka, Åsa Svedmark, Charlotte Häger

**Affiliations:** 1 Department of Community Medicine and Rehabilitation, Physiotherapy, Umeå University, SE-901 87, Umeå, Sweden; 2 Centre for Musculoskeletal Research, Department of Occupational and Public Health Sciences, University of Gävle, Gävle, Sweden

**Keywords:** Neck, Trapezius, Myalgia, Neck-shoulder pain, RCT, Individualized treatment, Rehabilitation, Physiotherapy, Tailored

## Abstract

**Background:**

A major problem with rehabilitation interventions for neck pain is that the condition may have multiple causes, thus a single treatment approach is seldom efficient. The present study protocol outlines a single blinded randomised controlled trial evaluating the effect of tailored treatment for neck-shoulder pain. The treatment is based on a decision model guided by standardized clinical assessment and functional tests with cut-off values. Our main hypothesis is that the tailored treatment has better short, intermediate and long-term effects than either non-tailored treatment or treatment-as-usual (TAU) on pain and function. We sub-sequentially hypothesize that tailored and non-tailored treatment both have better effect than TAU.

**Methods/Design:**

120 working women with minimum six weeks of nonspecific neck-shoulder pain aged 20–65, are allocated by minimisation with the factors age, duration of pain, pain intensity and disability in to the groups tailored treatment (T), non-tailored treatment (NT) or treatment-as-usual (TAU). Treatment is given to the groups T and NT for 11 weeks (27 sessions evenly distributed). An extensive presentation of the tests and treatment decision model is provided. The main treatment components are manual therapy, cranio-cervical flexion exercise and strength training, EMG-biofeedback training, treatment for cervicogenic headache, neck motor control training. A decision algorithm based on the baseline assessment determines the treatment components given to each participant of T- and NT-groups. Primary outcome measures are physical functioning (Neck Disability Index) and average pain intensity last week (Numeric Rating Scale). Secondary outcomes are general improvement (Patient Global Impression of Change scale), symptoms (Profile Fitness Mapping neck questionnaire), capacity to work in the last 6 weeks (quality and quantity) and pressure pain threshold of m. trapezius. Primary and secondary outcomes will be reported for each group with effect size and its precision.

**Discussion:**

We have chosen not to include women with psychological ill-health and focus on biomedical aspects of neck pain. Future studies should aim at including psychosocial aspects in a widened treatment decision model. No important adverse events or side-effects are expected.

**Trial registration:**

Current Controlled Trials registration ISRCTN49348025.

## Background

Neck pain, often combined with shoulder pain, is prevalent in working life but in most cases a specific cause for the pain is missing [1]. Hence, causal treatment will normally not be possible. Instead, general efforts to reduce symptoms are often used. Current best evidence of practice for chronic nonspecific neck-shoulder pain advocates a multimodal rehabilitation approach, which usually include psychological therapy, physical training, manual therapy and physiotherapeutic treatment [2-4]. However, knowledge on how to design the rehabilitation to achieve best effect is mostly missing, a viewpoint brought up by the Swedish Council on Health Technology Assessment [5]. A reasonable assumption is that the individual needs vary substantially due to different underlying pain mechanisms [1], and that the rehabilitation results for each patient will depend on the effects of the treatments of these mechanisms. This is certainly acknowledged in clinical practice today and common practice is to evaluate the individual patient and adjust the rehabilitation efforts to meet the need of each patient. However, no evidence based approach for such procedures have been reported.

Further, the evidence is rather modest for effects of specific single treatment approaches (for instance strength training, massage, manual therapy), or combinations of single treatments, despite a large increase in interventions studies addressing neck-shoulder pain in the last ten years [3]. This is probably due to wide group classification like nonspecific pain rather than identification of specific sub-populations or individual needs [6,7], which renders it less likely to achieve significant effects in single treatment intervention studies. At the same time, individual rehabilitation programs derived from assessment of each patient’s characteristics and needs, i.e., *tailored* rehabilitation [8], have not yet progressed nor been tested in controlled trials. To date, we are not aware of any study that evaluates tailored compared to non-tailored neck treatment programs consisting of the same treatment components. There are, however, studies comparing different treatments where the treatment of the individual patient is adapted to his or her condition (see e.g. [9,10]), although it is unclear how the treatments were individualized. Such pragmatic approaches are nearly impossible to repeat and thus of limited value for evidence based approaches.

In contrast to neck pain, tailoring or targeting treatment to subgroups that share specific characteristics has been applied for patients with nonspecific low back pain (LBP) [11,12]. For example, Brennan et al. [11] showed that targeted treatment based on sub-grouping according to standardized tests, clinical signs and symptoms leads to better outcomes in terms of improved disability, lower cost, and higher return-to-work rates compared to non-targeted treatment. However, a recent review indicates that results of the few existing studies comparing targeted to non-targeted treatment for LBP show only very cautious evidence of an advantage for targeted treatment [13].

Despite the above reasoning that single treatment modalities often show weak evidence in intervention studies for neck pain, there are several treatment methods that have shown positive effects on pain or functioning. These include strength training for neck-shoulder muscles [2,3,14], manual therapy [3,15], training of deep cervical flexor muscles [16-18] and eye-neck-hand coordination/proprioceptive training [17,19]. It seems feasible that several of these treatment components would be more effective when the decisions regarding their application are based on careful tailoring based on assessment of the specific dysfunctions. Therefore, such treatments should be considered as options in a clinical decision model provided that the model is based on valid assessments and theoretical rationale for application.

This study aims to contribute to the development of an evidence based clinical decision model for tailored rehabilitation of women with nonspecific neck-shoulder pain by testing the effects of tailored versus non-tailored treatment in a randomized controlled clinical trial (RCT). Our study focuses on women since they are known to be at a significantly greater risk for neck disorders than men [20]. An important feature of our decision model is that it is based on an extensive assessment of function, clinical signs and symptoms, with an attempt to apply clear cut-off values based on reference data combined with theoretical considerations. The assessment will be indicative to if a specific treatment should be applied or not. Our main hypothesis is that this decision model based tailored rehabilitation has better short, intermediate and long-term effects on primary and secondary outcomes than either non-tailored rehabilitation (same treatment components but applied quasi-randomly) or so called treatment-as-usual (TAU). We also hypothesize that tailored and non-tailored rehabilitation has a better effect than TAU (for details, cf. Current Controlled Trials registration ISRCTN49348025).

### Aims

The main aim of the study is to test the hypotheses described above by comparing the effects of tailored treatment, non-tailored treatment and treatment-as-usual for women with nonspecific neck-shoulder pain on physical function, pain intensity, overall improvement and satisfaction with treatment, work capacity, and other objective measures of functioning. A secondary aim is to evaluate the importance of physical and psychosocial factors in the workplace on long-term treatment outcomes. A third aim is to evaluate the cost-effectiveness of the treatment models from a health economics perspective.

## Methods/Design

### Design and setting

**Figure 1:**
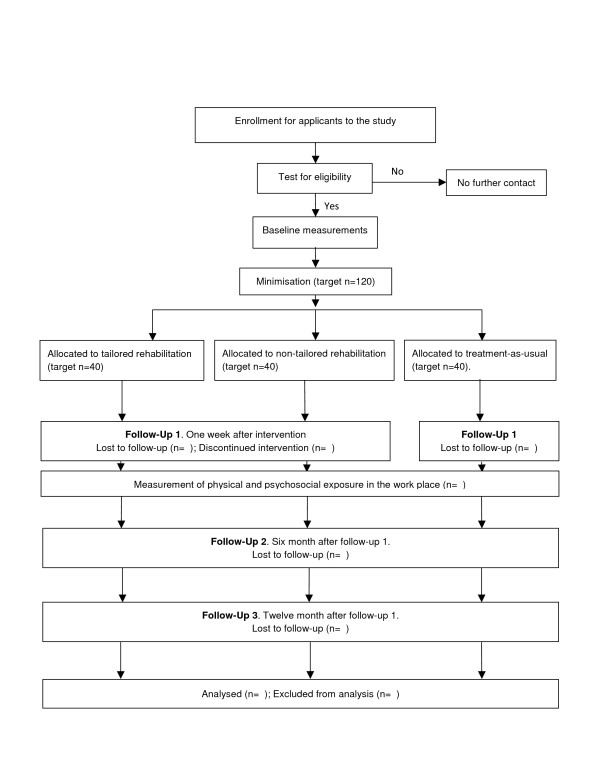
Flow-chart of research design.

The study is a prospective interventional single-centre, single-assessor, blinded randomized controlled clinical trial. Participants with neck-shoulder pain will be allocated in a 1:1:1 ratio to either tailored (T) or non-tailored (NT) treatment two to three times a week for 11 weeks (in total 27 sessions) or to treatment-as-usual (TAU). Minimisation [21,22] is used for the allocation sequence to minimise imbalance on the factors age, duration of pain, average pain intensity last week (Numeric Rating Scale – NRS) and disability due to neck pain (Neck Disability Index – NDI). Further, comparisons of baseline assessments will be performed with a control group without neck pain. The study will be executed in Umeå, Sweden. Assessment (questionnaires, laboratory and clinical assessments) of the participants with neck-shoulder pain is made one week before and after the treatment period, with follow-up measurements 6 and 12 month after end of treatment. The 12 month follow-up is only performed with questionnaires. See Figure 1 for a flow chart of the study. The study was approved by the Regional Ethical Review Board in Uppsala, Sweden (registration number 2011/081) and informed and written consent from participants is obtained according to the Declaration of Helsinki.

### Participants

The study sample will comprise of approximately 120 females, age 20–65, with a history of minimum six weeks of nonspecific neck-shoulder pain and 40 healthy controls matched on group level with respect to gender and age.

### Inclusion criteria

The inclusion criteria for the participants with pain (Table 1) are nonspecific neck-shoulder pain, defined as pain in the neck and surrounding tissues (indicated as dominant pain area in a pain drawing [23]) which include pain in the neck-shoulder muscles, excluding complaints related to the gleno-humeral joints. In addition to neck-shoulder pain, participants should have more than “no disability” but less than “complete disability” according to the Neck Disability Index (NDI) [24], and report impaired capacity on the quality or quantity to work the preceding month [25]. All participants with neck pain, as well as controls, should be Swedish speaking and understand written instructions in Swedish.

**Table 1:** Inclusion and exclusion criteria for participants with neck-shoulder pain

**INCLUSION**		
**Criteria**	**Reason**	**Assessment**
Neck-shoulder region as the dominant pain area	Neck pain is the focus area.	Pain drawing, clinical examination
Age 20-65	The study aim at the working population.	Questionnaire
≥ 10 and ≤ 68 NDI score	Focus on participants with mild, moderate or severe disability.	Questionnaire
Impaired capacity to work due to neck problems	Focus on participants with disability that is relevant for working life.	Questionnaire
**EXCLUSION**		
**Criteria**	**Reason**	**Assessment**
Trauma-related neck pain	Focus on nonspecific neck-shoulder pain.	Questionnaire
Cervical rhizopathy or vestibular dysfunction	Focus on nonspecific neck-shoulder pain without specific diagnosis that needs specific treatment.	Questionnaire, clinical examination at suspicion
Psychiatric, inflammatory, endocrinal, rheumatic, cancer, neurological or connective tissue disorders, stroke, heart infarct or type 1-diabetes	Focus on nonspecific neck-shoulder pain without specific diagnosis that needs specific treatment.	Questions if diagnoses exist from medical doctor
Concurrent low back pain	Predict poor treatment outcome and affect functioning.	Questionnaire
Fibromyalgia/generalized pain	Focus on nonspecific neck pain.	Questionnaire, clinical examination at suspicion
Low treatment expectation or catastrophizing most or all of the time	Prognostic factor for poor treatment outcome.	Questionnaire
Anxiety or depression	Prognostic factor for poor treatment outcome.	Questionnaire
Temporomandibular disorders	Focus on nonspecific neck-shoulder pain without specific diagnosis that needs specific treatment.	Questionnaire
Surgery or a fracture in the neck, back or shoulder, luxation of a shoulder joint	Risk that this affects the measurements in a specific way, unrelated to nonspecific neck pain.	Questionnaire
Severely restricted ROM in cervical rotation or shoulder flexion	Will not be able to accomplish the tests of functioning	Clinical examination

### Exclusion criteria

Exclusion criteria for the participants with neck pain are complaints related to trauma (questionnaire), cervical rhizopathy, vestibular dysfunction or other specific diagnosis such as psychiatric, inflammatory, endocrinal, rheumatic, cancer, neurological, or connective tissue disorders, stroke, heart infarct or type 1-diabetes (diagnosis from medical doctor). Specific clinical examination protocols [26,27] is used for confirmation at suspicion of rhizopathy (radiating symptoms below the shoulder, indicated on pain drawing), or vestibular dysfunction (rather strong/often dizziness or balance disturbances: ≥4 on both questions, scale 1–6 [28]). Participants are also excluded if they have concurrent LBP, which is a known predictor of poor treatment outcome in patients with neck pain [29,30] and may impair balance [31] and cervical motor function [32]. To be defined as having LBP, we use a case definition algorithm [33] which is based on the distribution in a cohort of 2,329 participants with a 5-year follow-up (for references, see [34,35]).

Further exclusion criteria are the following prognostic factors for poor treatment outcome; low treatment expectation [29,36], catastrophizing most or all of the time [29], anxiety or depression [37]. Low treatment expectation is assessed by the question *Do you think any kind of treatment or training will cure you?* with five response alternatives anchored by 1., “No, definitely not”, and 5., “Yes, make me completely cured”. Participants that answer according to response alternative 1 or 2 will be excluded. Catastrophizing is measured with a single question taken from the Pain Catastrophizing Scale [38]: *Have you felt your neck pain is terrible and that it’s never going to get any better?* Response alternatives are a 5-point ordinal scale (“all; most; some; little; none; -of the time”). The answers “most” or “all the time” indicates poor treatment outcome [29] and will lead to exclusion. Anxiety or depression is assessed by the Hospital Anxiety and Depression Scale with cut-off values of 10 for anxiety and 8 for depression [39]. Fibromyalgia/generalized pain is also an exclusion criterion and is assessed based on the diagnostic criteria of the American College of Rheumatology [40].

Participants with temporomandibular disorders are excluded. The criteria for exclusion are the answer “yes” on at least 2 out of 3 questions, combined with rating of the problem of ≥5 on a 11-grade scale (0 =”no problems”; 10 =”maximum problems”). The three questions are: *Do you have pain in the temple, face, jaw or jaw-joint once a week or more often? Does it hurt once a week or more often when you open your mouth or chew? Do you have locking in the jaw once a week or more often?* These criteria are based on Storm & Wänman [41].

Exclusion criteria are also if the participant has had surgery in the neck, back or shoulder, or fracture in the back or shoulder, the last 3 years. Also, fracture in the neck or thoracic spine, luxation of a shoulder joint the last year or severely restricted range of motion (ROM) in cervical rotation (< 30° in any direction) or shoulder flexion (< 110°) are exclusion criteria.

Participants belonging to the control group should not have ongoing problems or have had problems the latest 3 month in the neck or back and be generally healthy. Thus, the above mentioned exclusion criteria also apply to the control group. A summary of the inclusion and exclusion criteria for participants with neck pain is shown in Table 1.

### Recruitment procedures

Consecutive recruitment will be accomplished through the Occupational health service of Västerbotten County Council, Umeå municipality and Umeå University. Study invitations is announced on the web pages of these organizations and in local newspapers, or administered manually via staff members in the above organisations.

### Intervention leaders

There are four intervention leaders (ILs) who all are experienced physiotherapists (> 3 years) in the field of musculoskeletal disorders and with special education in manual therapy. Before the start of the study, 12 hours training for the therapists was accomplished involving principles of the study, attitudes towards the participants, the role of motor learning theory in the study and training in the treatment programs. All ILs will treat participants of both T- and NT-groups. Follow-up meetings between ILs and the project group will be held every second month throughout the intervention period.

### Treatment intervention

The intervention consists of treatment components and a decision model based on a number of tests for tailoring the treatment components to the individual participant. In the design of this model we first identified specific functional limitations or symptom based conditions for people with nonspecific neck pain through an extensive review of the literature. This resulted in five main categories of specific functional limitations or symptom based conditions:

1. Reduced cervical mobility

2. Impaired neck-shoulder muscle strength and motor control

3. Trapezius myalgia

4. Cervicogenic headace

5. Impaired eye-head-neck motor control

Visual impairment is also taken into consideration in the decision model since there is support for the notion that augmented activity levels in eye-muscles may cause a parallel increase in muscle activity in the neck-scapular area and cause fatigue, discomfort and pain [42].

As a second step we selected suitable tests to allow for characterizing the individual with respect to the categories. In the third step we selected treatments addressing these categories.

### Decision model for treatment

The decision model contains tests that should capture the specific functional limitations or conditions addressed in the intervention. The test results are then used for selection of treatment components for the intervention groups. The treatment components should be logically linked to the tests used. Our rationale when selecting tests, cut-off levels and treatment components have been the following:

Test are chosen which have the ability to detect impaired functions of importance in nonspecific neck-shoulder pain and that have good, or at least acceptable, re-test reliability.

We use tests and clinical assessments for diagnostics of certain defined neck conditions which have empiric and/or theoretic support for specific treatment (trapizius myalgia and cervicogenic headache).

Cut-offs used for the tests are based on empirical data from the literature or from our own reference data. In a few cases, we have adjusted the cut-off values according to theoretical and clinical considerations. With reference values at hand, the cut-off could be set either to give precedence to a high sensitivity or high specificity. We have chosen to prioritize a high specificity in the tests in an attempt to capture the impaired function in question and to avoid false positive outcomes. Further, we have chosen rigorous cut-offs at minimum ~ 20% below reference control values (see Table 2). This is based on the assumption that 20% difference is considered a clinical important difference (see e.g. Dworkin et al. [43]). We have also taken into account the relative number of positive tests predicted by our own reference data from a parallel study (ISRCTN trial registration number, ISRCTN92199001), avoiding to exceed >% of positive tests in order to keep the decision model diversified.

**Table 2:** Decision model for selecting tailored treatment

**Main factor**	**Test**	**Cut-off criteria**	**Rationale for cut-off**
**1. Cervical flexibility**	1.1 *Range of motion, upper cervical*		
Three sub-factors	a) Flexion-extension	a) < 68°	a) 20% below reference values of normative control data [44] resulting in 97% specificity.
	b) Passive rotation in maximal flexed position	b) < 32°	b) 18-29% below reference values of normative control data [45-47]. Also discriminating cut off for cervicogenic headache [47,48].
		**Qualifier**: Either a) or b)	
	1.2. *Range of motion, lower cervical*		
	Flexion-extension	< 17°	35% below reference values of normative control data [44] resulting in 94% specificity.
	1.3. *Range of motion, upper and lower cervical*		
	Axial rotation	< 109°	20% below reference values of normative control data [44] resulting in 97 % specificity.
**2. Cervical strength**	2.1.*Cranio-cervical flexion test*		
Three sub-factors	a) Maximal voluntary contraction (MVC)	a) < 2,5 Nm	a) Empirical experience, value indicating clear impairment (Shaun O’Leary, personal communication)
	b) Endurance (50 % MVC)	b) < 20 sec	b) 88,5% specificity based on normative control data (Pilot study, unpublished data, n = 26).
		**Qualifier**: Either a) or b)	
	2.2. *Cervico-thoracic test*		
	a) Flexion MVC	a) < 40 N	a) 95% specificity according to data simulation based on [49,50].
	b) Extension MVC	b) < 140 N	b) 95 % specificity according to data simulation based on [49,50].
		**Qualifier**: Either a) or b)	
	2.3. *Arm strength in lifting task*		
	a) Cervical Progressive Isoinertial lifting evaluation test (C-PILE) [51]	a) Max weight / adjusted body weight [52]: <0,12 kg/kg [28,53]	a) Cut off to discriminate between neck pain and healthy: Specificity 81% [53]
	b) Subjective rating of the ability to carry and to lift	b) At least answer “rather bad, rather difficult” on the questions “Because of your neck problems, how do you manage to carry/lift?” (≥4 on the scale 1 = Very good, no problem; 6 = Very bad, very difficult/impossibly)	b) Specificity data N/A. Chosen cut-off renders 47% of women with neck pain positive. (non-published data, ISRCTN92199001)
		**Qualifier**: a) and b)	Comment: From a clinical perspective, we thought it important to combine estimates of physical capability and subjective rating in the treatment decision process.
**3. Trapezius myalgia**	a) Diagnosed trapezius myalgia right or left	a) Criteria according to Ohlsson and coworkers [27], with amendments [26].	a) Specificity N/A. In an attempt to sharpen and objectify the trapezius myalgia criteria we have added pain pressure measurements.
	b) Pain pressure threshold of the upper trapezius muscles	b) < 175 N right trapezius, < 168 N left trapezius	b) 20 % below reference values of nonspecific neck pain subjects without trapezius myalgia.
		**Qualifier**: a) and b)	The combination of criteria predicts 40% positive tests (non-published data, ISRCTN92199001).
**4. Cervicogenic headache**	Diagnosed cervicogenic headache	Criteria of the Cervicogenic Headache International Study Group [54] with amendment of reduced range of motion specific for the upper cervical levels and palpable upper cervical joint dysfunction [55].	The reason for the amendment is to increase the sensitivity and specificity [55]. Note that the tests of reduced upper cervical range of motion are the same as for cervical flexibility 1a and b. [44,47,48]
**5. Sensorimotor control**	5.1. *Symptoms and activity limitations*	a) Rather strong/often dizziness or balance disturbances: (≥4 on both questions. Scale 1–6.) [28]	a) Prediction 11%.
Two sub-factors			
	Combinations of: - Dizziness or balance disturbances	b) Light dizziness or balance disturbances (3 on both questions, or >3 on one. Scale 1–6.) [28] and headache associated to neck problems (but not cervicogenic headache)	b) or c) Prediction 30%.
	- Headache associated to neck problems - Difficulties to rotate the head due to neck problems	c) Light dizziness or balance disturbances and, due to neck problems, difficulties to rotate the head	Disturbances of sensorimotor control and its associations to symptoms like dizziness/balance disturbances and headache is supported in the literature (for references see [56,57].
		(≥4 on scale 1–6.)	To predict the number of positive cases for the combinations in a), b) and c) we used 117 women with nonspecific neck pain (Own non-published data, ISRCTN92199001):
		**Qualifier**: Either a) or b)or c)	
	5.2. *Cervical motor function*	< 170°/sec	50% below reference control data giving 97% specificity [32]
	Peak speed of cervical axial rotation.		Reduced ability to perform fast cervical rotations may reflect altered sensorimotor function in neck pain patients [32].

MVC: Maximal voluntary contraction; C_PILE: Cervical Progressive Isoinertial lifting evaluation test.

The decision model including tests and cut-offs for selecting tailored treatment components is presented in Table 2. Immediately after the baseline assessment participants are allocated to one of the three groups; tailored (T), non-tailored (NT) and treatment-as-usual (TAU). For participants allocated to the T-group, the result of the decision model, a computerized decision algorithm based on the baseline assessment, is used as a basis for a preliminary selection of treatment components. The project leader (PL, author MB) conveys written preliminary treatment instructions to the IL. For the participants allocated to NT-group, two treatment components are quasi-randomly selected and exclude components that target the impaired function of the participant as revealed by the result of the decision model. Participants allocated to the TAU group do not receive any treatment from the study and no restrictions to what they are allowed to do. Participants in this group are told that they will be contacted after three months for a re-test.

In order to identify specific problems in terms of activity limitations due to the neck-shoulder pain and the significance of this limitation for the participant, the ILs interview each participant allocated to the T- group according to the Problem Elicitation Technique (PET) [58]. This is done at the first treatment occasion. The participant identifies six specific main problems together with the IL, and rate the difficulty and importance of each problem respectively, on 0–7 scales (higher scores mean more difficult/more important). Finally, the identified problems are ranked by the participant in terms of significance. The top three ranked problems are considered in the final composition of treatment components for the participant in the T-group as well as on the functional training of daily activities (see *Functional training of daily activities* below). After the PET interview, the IL and the PL meet to settle the final treatment direction. To this end, the outcome of the PET is considered together with the result of the decision model within predetermined rules (see *Rules for the decision model* below). Also, the prioritization order among treatment components is discussed if the participant has several (>3) in her treatment profile. The degree of impairment in relation to cut-off limits for each component as well as the outcome of the PET are considered if a priority order is set. Participants allocated to the NT-group are also interviewed at the first treatment occasion, but the questions included concern anamnesis and status focused on symptoms. The interview in both treatment groups is concluded by asking the participants what their personal goal with the rehabilitation would be. For the T-group, this goal should be connected to the specific activities listed in PET.

To identify participants that may have a need for optical correction and/or Visual Display Unit eye-glasses the following questions are asked: 1. *Do you wear spectacles, prescribed by a certified optician during the last 2 years, for near distance work?* 2. *Do you regularly perform visually demanding near-work, such as working with a computer or any other visually demanding near-work tasks, which requires a well functioning near vision?* If the answer is “yes” on the latter question, participants also answer 3. *Do you experience strain in or around your eyes at such work tasks?* The following combinations of answers qualify to be assessed by an optician: Participants ≥40 years of age; “No” on question 1 and “Yes” on the second question. Participants <40 years of age; “No” on question 1, “Yes” on question 2 and “Yes” on the third question. Only participants allocated to the T-group are sent to optician on qualification.

### Treatment components

The following treatment components were included (cf. corresponding factors and tests described in Table 2 as indicated by their numbers):

1. Manual therapy including mobilization treatment and training to promote range of motion, derived from the main factor *1. Cervical flexibility.* The three sub-factors shown in Table 2 (1.1, 1.2, 1.3) outline largely the cervical levels and the planes of motion to be treated. However, the specific cervical levels of manual mobilization/ROM-training as well as the target of structure (e.g. joint structures and/or extraarticular tissue like muscles and connective tissues) are decided by the therapist according to manual therapy principles [59]. Note that participants qualified according to the sub-factor 1.3 receive mobilization/ROM-training in rotation of the upper cervical only if cut-off 1.1.b. is reached.

2. Cranio-cervical flexion (CCF) exercise and strength training neck-shoulder-arm, derived from the main factor *2. Cervical strength*. The sub-factor 2.1 qualifies for specific CCF exercise program [60,61]. Sub-factor 2.2 entails moderate to high intensity strength training targeting neck-shoulder muscles. The program is inspired by Ylinen and co-workers [62] and the American College of Sports Medicine Position Stand [63], the latter regarding principles for movement speed, dose, load and frequency. 2.3 regards strength training for shoulder-arm muscles. The same training principles is used here as in 2.2.

3. EMG-biofeedback training, derived from factor *3. Trapezius myalgia*. The biofeedback treatment program consists of eight standardized exercises with gradual progression of difficulty level followed by training in specific tasks individualized for each participant in the tailored treatment group. For example, the specific task training could include wearing EMG-biofeedback equipment while working at a simulated computer-workstation with a stressful task. In this situation, postural corrections and re-education with the help of biofeedback may take place. The aim is to teach/train the participant to relax the upper trapezius muscles both in resting position and in static and dynamic tasks (to lower the contraction level of the trapezius muscle, obtain muscle relaxation in between contractions and to optimize the muscle strain during the task).

4. Treatment program for cervicogenic headache (factor 4 in Table 2). This is composed of manual therapy for the upper cervical and therapeutic exercise including CCF exercise and low-load endurance training for scapular muscles, mainly the lower trapezius and the serratus anterior, as well as postural correction of scapula in sitting. Thus, the treatment is adapted to current best evidence [64,65].

5. Neck coordination training / motor control training deriving from factor *5. Sensorimotor control*. There are two sub-factors delineated in Table 2: *5.1. Symptoms and activity limitations* and *5.2. Cervical motor function*. Participants qualifying for 5.1. receive a training program with exercise progressions of difficulty in three levels. The program is based on the work of Kristjansson and Treleaven [56,57] and consists of two main types of exercises: cervical repositioning/movement control and occulomotor exercises. The latter type consists of smooth pursuit, saccades, gaze stability and eye-head coordination exercises. Training of postural stability is usually an important component for patients with neck-shoulder pain and dizziness/unsteadiness. We have chosen to integrate this component in the other exercises at the third level of difficulty. Training following *5.2. Cervical motor function* focuses on improving the ability to perform fast cervical rotations. This may be done with partly the same exercises as in 5.1., but also in a dark room letting light flashes guide quick head movements in different movement planes and with varying trajectory length. In order to promote motor learning of the exercises including retention and transfer to other tasks and contexts, principles of motor learning theory [66] is part of the training program. For example, random practice in different contexts is introduced in order to enhance retention and transfer, and both internal (tactile, visual, proprioceptive) and external (verbally or by video) feedback is used to endorse the learning process.

### Rules for the decision model

Each participant allocated to the T-group should have at least two treatment components. If the outcome of the test algorithm only yields one or no treatment component, then the project group (PL and authors CH, MD) decides which component(s) that should be added. This decision is based on the relative closeness to cut-off for each test as well as the outcome of the PET. The same procedure is used if a treatment component is dropped for some reason (e.g., due to anatomical limitations for the treatment or other unforeseeable reasons as to why the treatment is not desired or feasible) in the beginning of the intervention period, and the participant thereby gets less than two treatment components. An exception to the rule of two treatment components is cervicogenic headache (4.). The treatment following this diagnosis consists of current best evidence [64,65], which involves both manual therapy and exercise therapy including cranio-cervical flexion exercise (see *Treatment components* above). Cervicogenic headache treatment program is only considered for participants in the tailored rehabilitation group.

A further component is chosen for T-group participants based on the same criteria if the decision algorithm yields any of the combinations of components as shown in Table 3

**Table 3:** Combinations of components that will lead to the addition of a further component

**Component 1**		**Component 2**
1.1. Range of motion, upper cervical	+	5.2. Cervical motor function
1.2. Range of motion, lower cervical	+	5.2. Cervical motor function
1.3. Range of motion, upper and lower cervical. Axial rotation.	+	5.2. Cervical motor function
5.1. Symptoms and activity limitations	+	5.2. Cervical motor function
1.1. Range of motion, upper cervical	+	1.2. Range of motion, lower cervical
1.1. Range of motion, upper cervical	+	1.3. Range of motion, upper and lower cervical. Axial rotation.
1.2. Range of motion, lower cervical	+	1.3. Range of motion, upper and lower cervical. Axial rotation.
1.1. Range of motion, upper cervical + 1.2. Range of motion, lower cervical	+	1.3. Range of motion, upper and lower cervical. Axial rotation.

These listed component combinations are excluded as treatment options in the NT-group. The rationale for excluding them is that these combinations of treatment do not suffice for the 11-week rehabilitation period with 27 treatment occasions.

A third treatment component could also be considered in the T-group if PET clearly indicates problems with activities that have close relation to a function/test included in the decision model, and the result of that test was close to cut-off for the specific individual.

If a participant experiences acute problems that negatively influence functions not included in her treatment component profile, the line of action from the therapist is first watchful waiting with adjustment of the participant’s current components during one week. If the problem remains after one week, the therapist is allowed to assess and treat with manual therapy maximally three times to reduce pain [3,15] irrespective of the participant’s treatment profile.

### Functional training of daily activities

In the latter half of the intervention period, functional training of daily activities is introduced and the treatment component program correspondingly reduced. For the participants of the T-group, each treatment component has predetermined suggestions for functional training exercises as a basis, but the idea is to individualize this part as far as possible. A meeting is held between IL and a person in the project group before commencing the training to discuss ideas for individual-specific functional training that targets the problematic activities pinpointed by the PET. Special concern is taken to organize the training according to principles of motor learning theory in order to enhance retention of the functional training tasks and transfer to new tasks and environments [66]. Thus, variation of the training/random practice is emphasized [67] with a gradual progression towards training in different contexts and increasingly more complex movement tasks, and external feedback is used. The functional training for the participants of the NT-group follows a set training program with complex movement exercises called “Muscle Action Quality (MAQ) training” [68]. The exercises are believed to enhance the general fitness qualities strength, flexibility, balance and movement control. The program is the same for all NT-group participants and includes movement exercises without weights and diagonal press- and pull exercises with weights. Thus, the exercises are not linked to specific daily activities as is the case for the T-group.

### Assessment

The baseline assessment has the following components and purposes: i) laboratory assessment including tests of motor control, strength and activity limitations, used for the treatment decision model as well as treatment outcome measures ii) clinical examination to categorize participants according to specific diagnostic criteria [26,27,55,69,70] used for the treatment decision model iii) clinical examination to confirm inclusion and exclusion criteria iv) questionnaires used for the treatment decision model and treatment evaluation. During a period of three months before commencing the study, the test leader was trained in performing assessments included in the test protocol. The test leader is blinded with regard to the group allocation of each subject.

### Laboratory assessment, test of functioning

The laboratory assessment described in this section correspond to factor 1 (*Cervical flexibility*), 2 (*Cervical strength*) and 5 (*Sensorimotor control*) in Table 2.

#### Cervical flexibility and Sensorimotor control

Cervical flexibility is measured in tests of active range of motion of the upper and lower cervical spine in flexion-extension and of axial rotations in normal upright posture. The procedures for these tests are identical to the descriptions given in [44]. Also, range of motion of axial head rotation during maximal forward neck flexion is measured. The test procedure is identical to that of Amiri et al. [45] except for that the axial rotations are passively imposed by the test leader and only two repetitions to each side (right/left) are performed. Cervical motor control is assessed in a test of maximal speed of cervical axial rotations [32]. All tests are performed in sitting and are given in the order in which they are described above. Kinematics of head movements relative to the thorax are measured with an electromagnetic tracking system (FASTRAK™, Polhemus Inc, USA) [45]. Outcome measures for the tests are maximum range of movement (degrees) and peak angular speed of the cervical axial rotations (degrees/second).

#### Cervical strength, cervico-thoracic test

**Figure 2:**
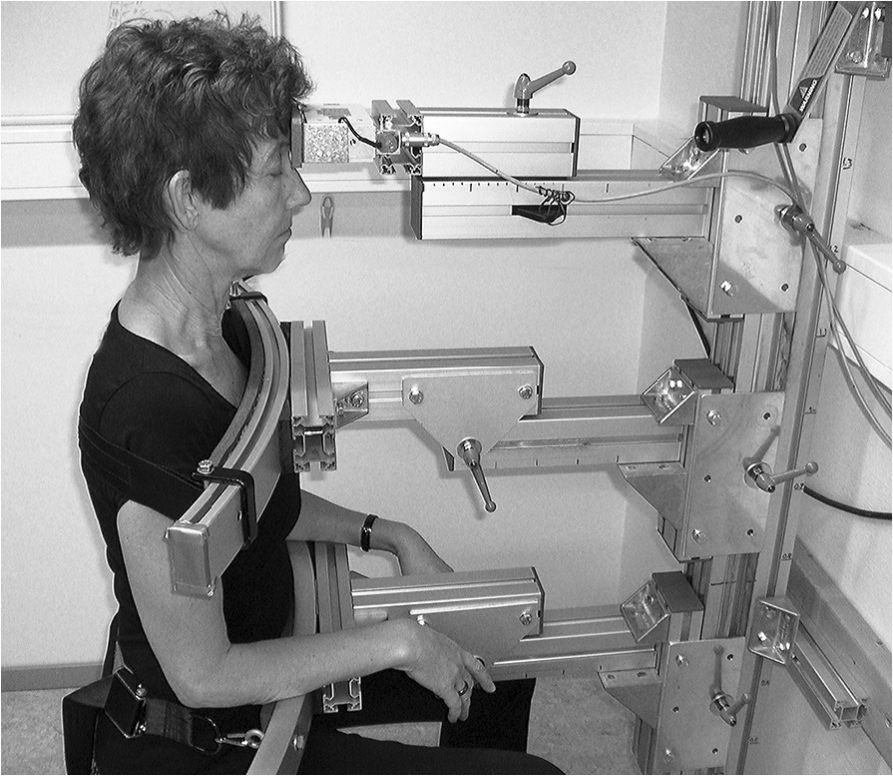
**Cervico-thoracic flexion test.** Sitting measurement of isometric cervico-thoracic flexion strength, maximal voluntary contraction (with permission).

Cervico-thoracic extension and flexion (Figure 2) maximal voluntary contraction (MVC) is performed in sitting with the same procedure as Salo and co-workers [49]. The strength values are expressed in Newtons.

#### Cervical strength, cranio-cervical flexion test

**Figure 3:**
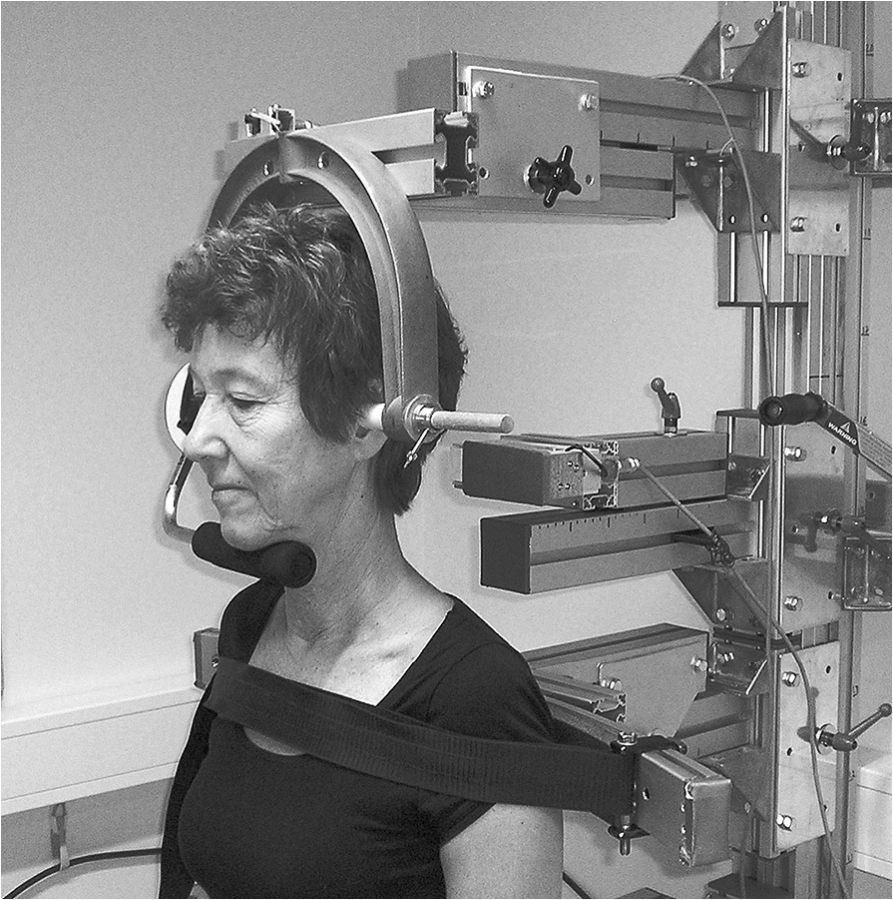
**Cranio-cervical flexion test.** Standing measurements of isometric cranio-cervical flexion strength, maximal voluntary contraction, and endurance 50% of maximal voluntary contraction (with permission).

The measurement device used for this test is based on O’Leary et al. [71], but in conformity with Van Wyke et al. [72] the test is performed in a standing position (Figure 3). In the starting position the dynamometer resistance arm of the measurement device is placed under the inferior border of the participant’s mandible. The dynamometer axis is aligned to the concha of the ear which corresponds to the axis of rotation for the atlanto-occipital joint over which the cranio-cervical flexor muscle group (the longus capitis and rectus capitis anterior muscles) acts. After getting accustomed to the device, instructions and a practice session in doing an isolated cranio-cervical flexion movement, the participant performs three maximum effort trials. If the last trial exceeds the second last by >5%, then a further trial is carried-out. Thereafter, the test leader explains and visualizes the endurance 50% MVC test with help of a computer screen showing the level of 50% MVC. The participant is asked to press against the resistance arm so that the torque curve reaches the 50%-level and to keep it there as long as possible. If the torque drops below the 50%-level, the test leader immediately instructs the participant to return to the level. The test ends either because of failure to keep the torque at the required level or when 10 accumulated seconds below the 50%-level bar has passed. The outcome of cranio-cervical flexion MVC test is torque (Newtonmeter) and the endurance 50% MVC test is time (seconds).

#### *Cervical strength*, *arm strength in lifting task*

The cervical progressive isoinertial lifting evaluation test (C-PILE) involves lifting weights placed in a box from waist height to shoulder height with progressively increasing loads [51,73,74].

### Clinical examination

#### Categorizing individuals with neck pain

Neck pain participants are examined by a physiotherapist according to a standardized physical examination protocol of the neck [27], with amendments presented by Juul-Kristensen and co-workers [26]. This protocol defines criteria for the diagnoses *Tension neck syndrome, cervicalgia, trapezius myalgia* and *cervical syndrome*.

#### Measurement of pressure pain threshold of neck muscles and pain provocation of neck facet joints

For quantification of muscle soreness in the upper trapezius muscles we use algometer pressure pain threshold (PPT) measurements. For acquaintance with the procedure, a first pressure pain test is performed distally of the left arm lateral epicondylia. Thereafter, the following measures are made: Right and left upper trapezius muscle, in the middle between processus spinosus of C7 and acromion, and a reference PPT in the tibialis anterior muscle of each leg. Three measurements are made on each spot, alternating between right and left side. The PPTs of the trapezius muscles are part of the decision model for treatment (see Table 2 and *Treatment components* above) as well as a secondary outcome measure (see below). Facets joints of the upper cervical spine are palpated for pain provocation and altered tissue resistance to movement as an indication of joint dysfunction [59]. The facet joint assessment is only performed on participants who are suspected to have cervicogenic headache (see below).

#### Assessment of specific diagnoses

The assessment for cervicogenic headache is based on the major criteria of Cervicogenic Headache International Study Group [54] with amendment of reduced ROM in the upper cervical segments and palpable upper cervical joint dysfunction [55]. Examination of rhizopathy [27] and vestibular dysfunction is made to comply with exclusion criteria. Participants who indicate pain in all four quadrants of the body in the pain drawing are examined for fibromyalgia/generalised pain [40]. Participants are excluded if the diagnosis is confirmed.

### Questionnaires

The following questionnaires are used for treatment evaluation: *General improvement*, the Patient Global Impression of Change scale (PGICS) [75]; *Pain intensity*, 0–10 Numeric Rating Scale (NRS) [75]; *Pain localization*, pain drawing [23]; *Physical functioning*, NDI [24], *Symptoms and functional limitations*, the Profile Fitness Mapping neck questionnaire (ProFitMap-neck), a neck specific questionnaire [28]; *Working capacity*, questions regarding the impact of neck symptoms on the quantity and quality of performed work [25]. Self-estimated improvement during treatment is measured after 10 and 20 treatment sessions with PGICS and NRS.

For measurement of *health economy* EuroQoL 5-dimensions (EQ-5D) [76] and the Short Form Health Survey (SF-36) [77], reflecting health-related quality of life, are used. In addition, we apply measures of the cost of intervention, self-reported absence from work due to neck pain, prescription and consumption of drugs and health care, respectively.

Work load exposure is assessed by questionnaires and observations. *Psychosocial factors* are estimated by selected scales from the General Nordic Questionnaire for Psychological and Social Factors at Work (QPS Nordic) [78]: Quantitative job demands; Decision demands; Learning demands; Control of decisions; Control of work pacing; Perception of mastery; Support from superior; Support from coworkers; Support from friends and relatives; Social climate. We also use a question on perceived stress with a 5-point response scale [79]. Finally, *Physical factors* are assessed by observations in the workplace (Quick Exposure Check) [80].

All participants allocated to the T- or NT-groups are also followed qualitatively during the intervention period through standardized as well as open ended questions after 10, 20 and at the last treatment session, respectively.

### Primary outcome measures

1. Physical functioning, measured with the Neck Disability Index (NDI)

2. Average pain intensity last week, measured with the Numeric Rating Scale (NRS)

### Secondary outcome measures

1. General improvement, assessed by the Patient Global Impression of Change scale (PGICS)

2. Symptoms, measured by the symptom index of the Profile Fitness Mapping neck questionnaire (ProFitMap-neck)

3. Capacity on the quality and quantity to work in the latest 6 weeks due to neck problems ([1 – (quality/10) × (quantity/10)] × 100%)

4. Pressure pain threshold of m. trapezius, assessed with pressure algometer measurement

### Other outcome measures

1. Self-estimated improvement during treatment, for General improvement, “much improved” or “very much improved” on PGICS; for Pain intensity, no pain last week (NRS)

2. Functional limitations and compound total score, assessed by the Profile Fitness Mapping neck questionnaire (ProFitMap-neck)

3. Cervical range of motion (active flexion-extension; active axial rotation; passive flexion-rotation)

4. Peak speed in cervical axial rotation

5. Cranio-cervical flexion endurance (50% of maximum voluntary contraction)

6. Lifting capacity, assessed by the cervical progressive isoinertial lifting evaluation test (C-PILE)

7. Physical activity, measured with LIV 2000

8. Health-related quality of life for clinical and economic appraisal, assessed by the EQ-5D

9. Questions on reporting sick, and consumption of care

10. Quality of life - Mental health, assessed by the Mental component summary in the SF-36

11. Quality of life – Physical Health, assessed by the Physical component summary in the SF-36

12. Area of pain distribution, assessed by pain drawings

13. Adverse events. Open ended questions, and PGICS administered after 10, 20 and the last treatment session. “Much worse” or “Very much worse” on the PGICS is equalized with an adverse event.

### Statistical methods

#### Power

Power calculations with the one-way analysis of variance (ANOVA) routine (nQuery Advisor 3.0) are presented with regard to treatment effects for the primary outcome measures Physical functioning, and Average pain intensity last week. For physical functioning measured with NDI, a clinical important difference is between 6–10 NDI% [81]. Reference data from a parallel clinical trial (ISRCTN92199001) showed that the NDI standard deviation (SD) was 10.3 NDI% (based on 117 women with neck-shoulder pain). To obtain a power of 0.8, given a difference of 6 NDI% between any of the three groups, 20 individuals are required in each group (alfa = 0.05). For the average pain intensity last week, the smallest clinical important pain reduction measured with NRS is approximately 15% [43]. In the above mentioned clinical trial, the SD was 15.5 NRS%. Given these facts, 20 individuals in each group is sufficient to obtain a power of >0.8 for a difference of 15 NRS% between any of the three groups (alfa = 0.05). On the basis of this power calculation we aim at recruiting 40 participants for each group, which would give us a satisfactory safety margin to retain a power of 0.8 or higher through the trial.

#### Data analyses

Statistical analyses will be performed with IBM SPSS statistics version 20.0 and the level of significance is set at p < 0.05. Primary and secondary outcome variables will be analysed according to intention-to-treat (ITT) and per protocol (PP). In ITT analyses, all participants allocated to the study groups constitute the study sample, whereas only participants that complete a minimum of 14 treatment sessions (≥50% of the total number of treatment sessions) and have valid measurements at baseline and follow-up will be the study sample in the PP analyses. In analyses of the main hypothesis, effect size and the treatment effect (between-group mean differences and 95% CI) will be determined for the primary and secondary outcome variables by means of analysis of covariance (ANCOVA) utilising mixed model approach, with treatment as fixed factor and baseline measurement of the outcome variable as covariate. If the residuals of an ANCOVA model are not normally distributed, appropriate transformations of relevant variables will be used in the analysis. Full model for post hoc tests will be used. For the variable General Improvement (PGICS) comparisons between the groups will be made by proportional odds model (ordinal logistic regression) and estimation of the absolute effect (risk difference) with 95% CI.

Analyses regarding the secondary aim to evaluate the importance of physical and psychosocial factors in the workplace on long-term treatment outcomes will be handled in the following way. Participants that show an improvement exceeding minimal clinically important difference for the primary outcomes (6 NDI% or 15 NRS%), at follow-up 1 (one week after intervention), will be classified as “improved”. Long term effects (measured at 12-month follow up) of workload factors on primary outcome variables and the secondary variable Capacity on the quality and quantity to work will be tested for the group “improved” with General Linear Model ANCOVA where outcome values at follow-up 1, as well as the factors for work load exposure (see *Questionnaires*), are covariates. Exposure of workload is measured 1 week after end of intervention. At 6-month and 12-month follow-up participants answer questions on if exposure has changed the latest 6 month. Those whose self-rated physical or psychosocial work exposure markedly changed (measured with 2 questions) between follow-up 1 and 12-month test will be excluded for further analyses related to the secondary aim.

The third aim to evaluate the cost-effectiveness in the groups, cost-utility analyses (CUA) comparing measures of health gain and measures of costs will be performed [82]. For this purpose, estimates of quality adjusted life year (QALY) gains based on EQ-5D will be calculated, followed by cost by QALY ratio.

## Discussion

We delimit the study to participants without psychological ill-health to avoid the interventions and the decision model to get too diversified and complex. We acknowledge, however, that psychological ill-health is a common co-morbidity to chronic neck pain. Future studies should therefore aim at including this aspect in a widened treatment decision model. Our study focuses on women since women are known to have a significantly greater risk for neck disorders than men [20]. This means, however, that the results of the study cannot readily be extrapolated to men. Finally, no adverse events or side-effects are expected from the tests or treatments given in the study.

## Abbreviations

ANCOVA: Analysis of covariance; ANOVA: Analysis of variance; CCF: Cranio-cervical flexion; C-PILE: Cervical progressive isoinertial lifting evaluation test; CUA: Cost-utility analyses; EMG: Electromyography; EQ-5D: EuroQoL 5-dimensions; IL: Intervention leader; ITT: Intention-to-treat; LBP: Low back pain; MAQ: Muscle Action Quality; MVC: Maximal voluntary contraction; NDI: Neck Disability Index; NRS: Numeric Rating Scale; NT-group: Non-tailored treatment group; PET: Problem Elicitation Technique; PGICS: Patient Global Impression of Change scale; PL: Project leader; PP: Per protocol; PPT: Pressure pain threshold; ProFitMap-neck: Profile Fitness Mapping neck questionnaire; QALY: Quality adjusted life year; QPS Nordic: General Nordic Questionnaire for Psychological and Social Factors at Work; RCT: Randomized controlled clinical trial; ROM: Range of motion; SF-36: Short Form Health Survey; TAU: Treatment-as-usual; T-group: Tailored treatment group.

## Competing interests

The authors declare that they have no competing interests.

## Authors’ contribution

MB conceived of the study, design, planning and drafted the manuscript. MD conceived of the study, design, planning and helped to write the manuscript. ÅS conceived of the study, design, planning and helped to write the manuscript. CH conceived of the study, design, planning and helped to write the manuscript. All authors read and approved of the final manuscript.

## Pre-publication history

The pre-publication history for this paper can be accessed here:

http://www.biomedcentral.com/1471-2474/13/75/prepub
